# A Localized Surface Plasmon Resonance Sensing Method for Simultaneous Determination of Atenolol and Amiloride in Pharmaceutical Dosage Forms and Urine Samples

**DOI:** 10.1155/2018/9065249

**Published:** 2018-01-17

**Authors:** Marwa R. El-Zahry

**Affiliations:** Pharmaceutical Analytical Chemistry Department, Faculty of Pharmacy, Assiut University, Assiut 71526, Egypt

## Abstract

This contribution describes a simple, fast, and sensitive application of localized surface plasmon resonance effect of silver nanoparticles for simultaneous determination of antihypertensive drugs' mixture atenolol and amiloride in both pharmaceutical dosage forms and in biological samples (urine). Silver nanoparticles were prepared by chemical reduction of silver nitrate using hydroxylamine HCL in an alkaline medium. Application of silver-hydroxylamine nanoparticles (SH NPs) provides many advantages including reproducibility, sensitivity, and cost effective way of analyte determination. Amiloride has four amino groups which act as attachment points on the surface of silver nanoparticles resulting in a synergistic effect on the absorption intensity of atenolol, leading to increase the sensitivity of the determination of both compounds. This method shows excellent advantages comparing with the previously reported methods, including accuracy, precision, and selectivity. The linear range of atenolol is 1 × 10^−5^–1 × 10^−4^ mol·L^−1^ and of amiloride is 1 × 10^−6^–1 × 10^−5^ mol·L^−1^. The limit of detection (LOD) values of atenolol and amiloride are 0.89 × 10^−5^ and 0.42 × 10^−6^ mol·L^−1^, respectively.

## 1. Introduction

Atenolol (ATN) is a *β*_1_-selective adrenoreceptor antagonist, a drug belonging to the beta blockers. ATN is used primarily in cardiovascular diseases either alone or concurrently with other antihypertensive drugs because of their additive effects in the treatment of myocardial infarction, arrhythmias, and angina. It decreases heart rate and the force of the heart muscle contraction and reduces blood pressure by blocking the action of the nervous system on the heart [1].

Amiloride (AML) is a mild potassium-sparing diuretic that acts by inhibiting sodium-potassium ion exchange through blocking the distal renal tubule of the nephron [2].

Literature survey reveals that few analytical methods have been reported for the simultaneous determination of ATN and AML in pharmaceutical dosage forms and in some biological samples, for example, urine, blood, and plasma. RP HPLC method was applied for simultaneous estimation of both analytes in the presence of hydrochlorothiazide [3]. HPLC and chemometric-assisted spectrophotometric methods were reported for determination of ATN, AML, and chlorothalidone [4]. ATN and AML were simultaneously investigated in their pharmaceutical tablets using capillary zone electrophoresis [5, 6]. Simultaneous resolution of ATN, AML, hydrochlorothiazide, and timolol was studied employing different chemometric methods [7]. Different antihypertensive drugs including ATN and AML were determined using first-derivative nonlinear variable-angle synchronous fluorescence spectrometry [8].

Silver nanoparticles (AgNPs) have been the focus of research for many years because of their interesting optical properties [9, 10]. When AgNPs are scattered in liquid media, they exhibit a strong UV-visible extinction band which is not present in the spectrum of the bulk metal. This extinction band results when the incident photon frequency is resonant with the collective excitation of the conduction electrons and is known as the surface plasmon resonance (SPR) [11–14]. The plasmon resonance absorption of AgNPs has a molar extinction coefficient (3 × 10^11^ M^−1^·cm^−1^) [15], which allows higher sensitivity in optical detection methods than conventional reagents.

Localized surface plasmon resonance (LSPR) is a type of surface plasmon excitations. It happens when light hits a nanoparticle having a size smaller than the wavelength of the incident light. The incident photon may combine with the metal electrons and begin to oscillate coherently [16, 17]. As LSPR is sensitive to material type, size, and dielectric constant, it is considered to be a powerful technique that has been used recently in biosensing and biomedical applications [18, 19].

Moreover, LSPR is responsible for enhancing the surface sensitivity of different spectroscopic methods including fluorescence [20] and Raman scattering [21, 22]. The reported applications of LSPR found in literature are based on the optical detection and its enhancement such as in surface enhanced Raman spectroscopy (SERS) which is applied for molecule detection using the extinction of metal nanoparticles [23–25].

After careful survey, it has been concluded that this research is considered the first effort for the simultaneous quantitative determination of ATN and AML in laboratory-made mixtures, pharmaceutical dosage forms, and in biological fluids (urine) using LSPR produced from SH NPs.

This approach is based on the adsorption interaction between the studied analytes and the synthesized AgNPs resulting in enhancement effect in the UV absorption intensities of the mixed analytes. This enhancement provides a more sensitive and accurate quantitative tool for analytical applications.

## 2. Experimental

### 2.1. Chemicals

Silver nitrate, hydroxylamine HCL, sodium hydroxide, and the essential chemicals for preparation of SH NPs were purchased from Sigma-Aldrich. Potassium nitrate and potassium chloride were purchased from Sigma-Aldrich and used to adjust the ionic strength. ATN and AML compounds were generously donated from EPICO Co, Cairo, Egypt. Distilled water was used for the preparation of all solutions throughout the experiment. Atenoretic® capsules were purchased from the local market, containing 50 mg ATN and 2.5 mg AML.

### 2.2. Instruments

The extinction spectra were recorded using a computerized, UV-visible spectrophotometer (UV-1601 PC, Shimadzu, Japan) with 1.0 cm quartz cells.

Characterization of the synthesized SH NPs was done using a transmission electron microscope (TEM; FEI TECNAI F20) and a scanning electron microscope (SEM; FEI Quanta 200 FEGSEM).

### 2.3. Synthesis of SH NPs

SH NPs were prepared following the procedure described by Leopold and Lendl [26] using hydroxylamine HCL in an alkaline medium as a reducing agent for chemical reduction of silver nitrate solution. The concentration of silver nitrate in the prepared solution mixture was set to be 1 × 10^−3^ mol·L^−1^. A solution mixture of 6 × 10^−2^ mol·L^−1^ hydroxylamine HCL and 0.1 mol·L^−1^ NaOH was rapidly added to the silver ion solution with shaking until milky grey color colloids were obtained.

The pH tends to decrease with time because of the formation of nitrogen dioxide as a gaseous reaction product. Therefore, sodium hydroxide solution (0.1 mol·L^−1^) was added to the solution mixture to adjust the pH value. The pH value of the applied SH NPs is 6.0, as the prepared colloids were aged for one day prior to its use. The calculated molar concentration of the prepared AgNPs was 5 × 10^−7^ mol·L^−1^ [27].

### 2.4. Simultaneous Determination of ATN and AML

In a typical procedure for determining the studied analytes, 100 *µ*L of the prepared SH NPs, 1.0 mL of 50 mmol·L^−1^ KNO_3_ solution, and different concentrations of both ATN and AML in mixture were transferred into a 10 mL volumetric flask. Then the mixture was diluted to the mark with distilled water to reach the final concentrations of the investigated drugs that lie under the studied calibration ranges. Finally, the reaction solution was transferred into a 1 cm spectrometric cell to record the absorption spectra of the studied concentrations of ATN and AML. The calibration curves of both analytes were constructed using the absorbance enhancement at 274 nm for ATN and 364.5 nm for AML. A blank experiment was also carried out under the same conditions without adding the studied analytes.

### 2.5. Applications of the Proposed Method

The content of ten capsules was obtained, and the average weight of one capsule's content was weighed and accurately transferred into a 100 mL volumetric flask containing distilled water. The flask content was sonicated for 30 min and then completed to the mark with distilled water. After filtration, one milliliter of the clear filtrate was taken, and the procedure was conducted as previously described. In the case of urine sample analysis, the urine sample was diluted 50 times before the application to minimize the effect of the complicated matrix. The procedure was conducted as described in Experimental.

## 3. Results and Discussion

### 3.1. Characterization of SH NPs

**Figure 1:**
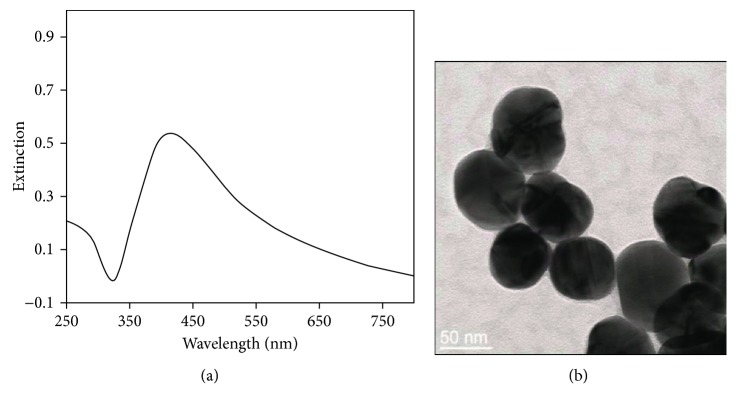
(a) Extinction spectrum of SH NPs. (b) TEM image of the prepared SH NPs.

In order to characterize the prepared colloids, TEM images were recorded for the prepared SH NPs.  Figure 1 shows the extinction spectrum of SH NPs after dilution of the prepared colloids ten times with distilled water. The maximum extinction is located at 416 nm which is in agreement with the reported approach suggested by Leopold and Lendl [26]. The full width at half maximum (fwhm) is the indication of the particle dispersion. It has been calculated to be 166 nm which indicated the polydispersion of the particles.

### 3.2. Synergistic Effect Based on LSPR of AML and ATN

Since pKa values of ATN and AML molecules are 9.7 and 8.6, respectively, and because the SH NPs have pH 6.0, the protonated form of both analytes will be considered.

SH NPs with a negative charge in their surface are able to distribute from each other through the electrostatic repulsion. In the case of ATN, it was previously reported that the nitrogen atoms in the amino groups have more positive charges, while the negative charge is mainly located on oxygen atoms and on aromatic ring [28]. Different from the ATN molecule, AML contains four amino groups. These groups can make the molecule to carry a positive charge at a specific pH, enabling AML from adsorption onto the negatively charged surface of SH NPs through the electrostatic attraction [29, 30].

**Figure 2:**
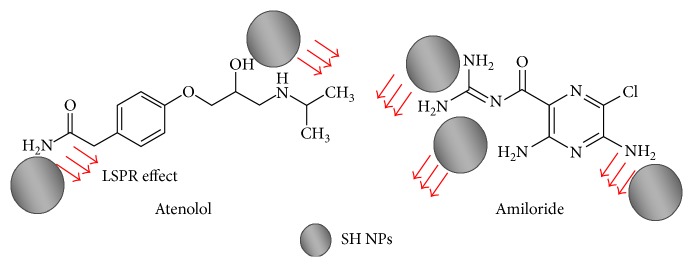
Proposed mechanism of the interaction of ATN and AML on SH NP surface.

Figure 2 describes a scheme suggesting the mechanism of interaction between the studied analytes and SH NPs.

**Figure 3:**
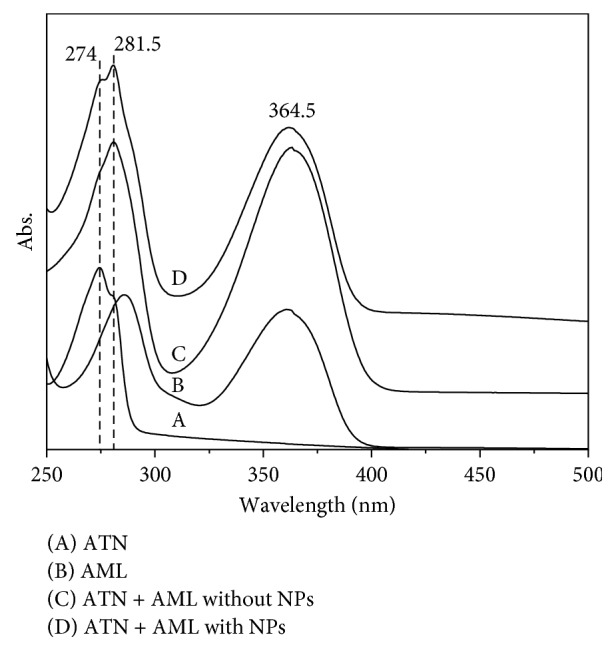
(A) UV-visible spectra of 1 × 10^−4^ mol·L^−1^ ATN, (B) 5 × 10^−6^ mol·L^−1^ AML, and (C) ATN and AML mixture in absence and (D) in presence of 100 *µ*L SH NPs and 50 mmol·L^−1^ KNO_3_. (The spectra were shifted vertically for clarification.)

In aqueous solution, ATN and AML show absorption band at 281 and 364.5 nm, respectively (Figure 3). Upon addition of ATN and AML molecules to the prepared SH NPs, one new band appeared at 274 nm for ATN. The appearance of the absorption bands of the drugs and the disappearance of absorption peak of SH NPs may be attributed to the adsorption of the drugs' molecule containing six amino groups totally on the silver nanostructures surface. This phenomenon had been seen in studying the interaction of plasmon and molecular resonance for R6G and AgNPs [31].

From Figure 3, we can notice two main points. The first one is the enhancement effect of the absorption intensity of the studied analyte mixture upon adsorption on the surface of SH NPs. The enhancement effect of ATN plasmon band provides a sensitive determination of ATN in the presence of AML. The second point is the appearance of an absorption band at 274 nm which is attributed to light absorption of ATN; while in the case of absence of SH NPs, this band is not distinguished. So, by adding the prepared colloids as a substrate for the analysis of ATN and AML mixture, this will provide an accurate and sensitive simultaneous quantitative determination in laboratory-made mixtures, pharmaceutical dosage forms, and in urine samples.

### 3.3. Optimization of Experimental Parameters

The ability of SH NPs to enhance the absorption spectrum of the studied mixture is greatly affected by different experimental parameters such as pH, ionic strength, and SH NP concentration. So, each parameter was studied and optimized to establish the analytical performance of the approach, while keeping other factors constant.

#### 3.3.1. Effect of pH

**Figure 4:**
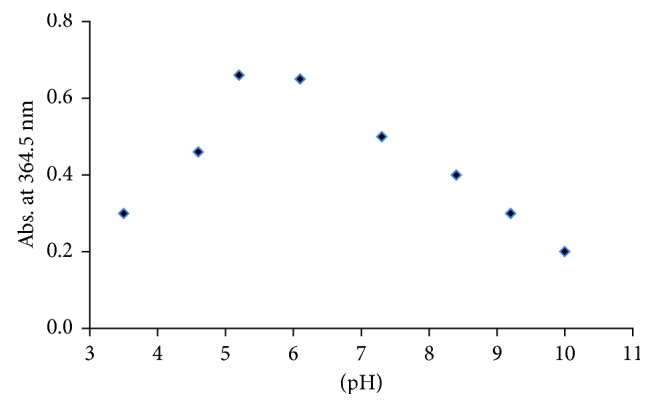
Effect of pH on the absorption intensity of 1 × 10^−4^ mol·L^−1^ ATN and 5 × 10^−6^ mol·L^−1^ AML mixture. Experimental conditions: 100 *µ*L SH NPs and 50 mmol·L^−1^ KNO_3_.

The interaction between the prepared SH NPs and the studied analytes is significantly affected by the pH value of the solution. The pH may alter the surface charge of the colloids and hence may interfere the interaction with the analytes. Considering a mixture of 1 × 10^−4^ mol·L^−1^ ATN and 5 × 10^−6^ mol·L^−1^ AML, the absorption intensity at 364.5 nm as a representative example was measured against different pH values. It is important to select the optimum pH value that gives the maximum intensity at 364.5 nm. So, pH ranging from 3 to 10 was investigated by adjusting the pH value of SH NPs using 0.1 mol·L^−1^ NaOH and 0.1 mol·L^−1^ H_3_PO_4_ solutions. The optimization results of the pH values are presented in Figure 4, and it can be seen that the highly acidic or highly basic conditions have negative effect on the absorption intensity via decreasing the absorbance value at 364.5 nm which is not required. While the maximum intensity was obtained when the pH was ranged from 5 to 6, it was selected as an optimum pH value throughout the whole experiments. This result is agreed with the previously studied research that suggested that the electrostatically stabilized nanoparticles are greatly affected by the change in pH [32].

#### 3.3.2. Effect of Type and Ionic Strength of Aggregating Agent

**Figure 5:**
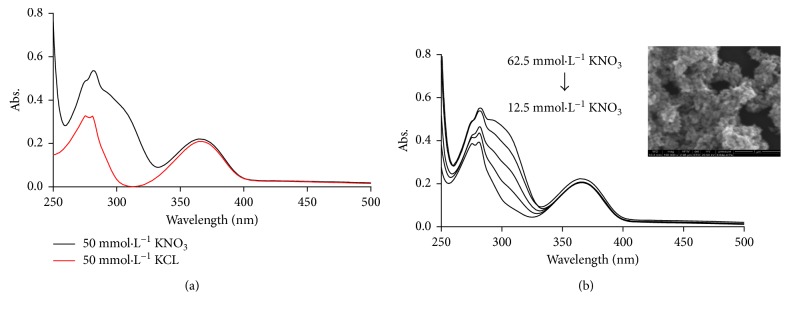
(a) Effect of electrolyte type and (b) effect of different concentrations of KNO_3_ on the absorption intensity of 1 × 10^−4^ mol·L^−1^ ATN and 5 × 10^−6^ mol·L^−1^ AML mixture in the presence of 100 *µ*L SH NPs. Inset: SEM image of SH NPs after addition of KNO_3_ (50 mmol·L^−1^).

The type of electrolytes as the aggregating agent has been tested (KNO_3_ and KCL solutions) to obtain the optimum type that will be used throughout the whole experiment. From Figure 5, we can conclude that the addition of nitrate ions enhanced the aggregation of SH NPs rather than chloride ions. It is reported that the aggregating agent may compete with the analyte for the surface of the colloid.

This phenomenon was explained previously [33], and it was reported that nitrate anions as aggregating agent are more easily displaced than chloride anions, and the effect of ionic strength was optimized by addition of different concentrations of potassium nitrate solution to the prepared SH NPs and studied their effect on the absorption intensity of the mixed analytes (1 × 10^−4^ mol·L^−1^ ATN and 5 × 10^−6^ mol·L^−1^AML). Potassium nitrate concentrations were ranged from 12.5 to 62.5 mmol·L^−1^. Figure 5 shows that the more intense absorbance can be obtained when more concentrated KNO_3_ solution was used, until it reached 50 mmol·L^−1^, and then no further significant absorbance increase is found.

Thereby, 50 mmol·L^−1^ KNO_3_ was selected as an optimum concentration of the electrolyte solution. For further confirmation of the aggregation effect of KNO_3_ on SH NPs, a SEM image was monitored for SH NPs after addition of 50 mmol·L^−1^.

#### 3.3.3. Effect of SH NP Concentration

**Figure 6:**
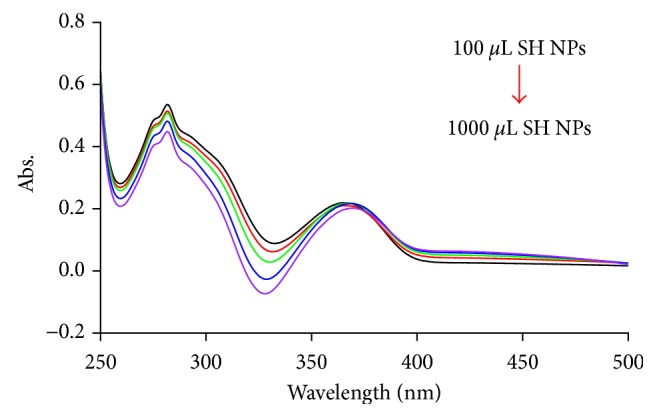
Effect of SH NP volume on the absorption intensity of 1 × 10^−4^ mol·L^−1^ ATN and 5 × 10^−6^ mol·L^−1^ AML mixture in the presence of 50 mmol·L^−1^ KNO_3_.

SH NP concentration has a significant effect on the interaction of NPs with ATN and AML molecules. The absorption spectra of the reaction mixture with a variety of SH NP volumes are given in Figure 6. The studied volumes of the prepared SH NPs were ranged from 100 to 1000 *µ*L using 1 × 10^−4^ mol·L^−1^ ATN and 5 × 10^−6^ mol·L^−1^ AML in mixture and 50 mmol·L^−1^ KNO_3_ as an aggregating agent. As it is shown, high intensities and hence high sensitivity were obtained with a low NP volume. Thereby, 100 *µ*L of SH NPs was selected to be the optimum volume throughout the whole experiments.

### 3.4. Validation of the Proposed Method

#### 3.4.1. Linearity

**Figure 7:**
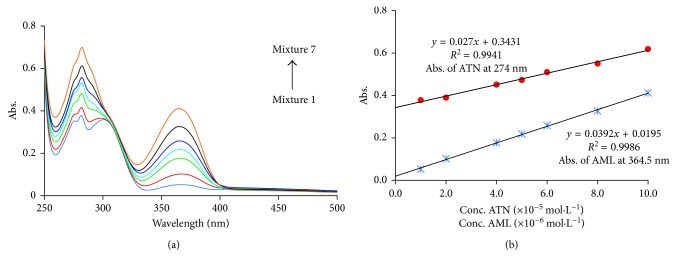
(a) Calibration spectra of different concentrations of ATN and AML mixture. From 1 to 7 mixtures, they are 1, 2, 4, 5, 6, 8, 10 × 10^−5^ mol·L^−1^ ATN with 1, 2, 4, 5, 6, 8, 10 × 10^−6^ mol·L^−1^ AML, respectively. (b) Linear relationship curves of ATN (measured at 274 nm) and AML (measured at 364.5 nm) using 100 *µ*L SH NPs and 50 mmol·L^−1^KNO_3_.

Figure 2 shows the interaction between SH NPs and ATN molecule. In the case of AML, the interaction points with SH NPs are four protonated amino groups. This explains the high sensitivity of AML's determination (1 × 10^−6^–1 × 1 0^−5^ mol·L^−1^) rather than the sensitivity of ATN (1 × 10^−5^–1 × 10^−4^ mol·L^−1^).  Figure 7 shows the spectral change of seven absorption spectra with increasing the concentration of the mixed analytes. Two calibration curves of the mixed solutions of ATN and AML were constructed (three replicates for each reading) by recording the absorption intensities at 274 nm for ATN and 364.5 nm for AML (Figure 7). The statistical parameters required for the evaluation of linearity of the developed method as stated by ICH guidelines [34] are summarized in Table 1.

For further evaluation of the linearity, the Mandel fitting test [35] was done, where at 95% confidence level, the critical *F* value is equal to 7.7. As the calculated *F* values (7.8 × 10^−9^) for ATN and (7.2 × 10^−8^) for AML are less than the critical *F* value, indicating that the linear regression is the best way to describe this relationship.

#### 3.4.2. Precision and Accuracy

The precision of the developed method was evaluated by testing the intraday precision by analysis of six replicate separate solutions of the working standard mixtures of ATN and AML at three different concentration levels for each analyte. Interday precision was examined by repeating the analysis of the studied mixture over a period of three consecutive working days. The precision results are calculated as RSD% which was found to be ≤3.1 indicating good repeatability and reproducibility of the proposed method (Table 1).

#### 3.4.3. Selectivity

In order to investigate the selectivity of the proposed method, the general procedure was followed in the presence of 1 × 10^−4^ mol·L^−1^ ATN and 5 × 10^−6^ mol·L^−1^ AML with some common interfering ions such as (ascorbic acid, Ni^2+^, Na^+^, Ca^2+^, NH_4_^+^, SO_4_^2−^, and PO_4_^3−^) at a concentration of 1 × 10^−3^ mol·L^−1^. From the obtained results in Table 2, it can be indicated that the proposed method has no interference even in the presence of high concentrations of other interfering ions.

#### 3.4.4. Robustness

It was examined by evaluating the influence of small variation of method variables including; concentration of SH NPs, concentration of KNO_3_ and the volume taken of the studied analytes on the method suitability and sensitivity. Results are shown in Table 3. It was found that none of these variations affect the method significantly, indicating the reliability of the proposed method during normal usage and so the proposed method is considered robust.

#### 3.4.5. Application of the Proposed Method

The developed method was applied for the determination of the investigated analytes in laboratory-prepared mixture by varying the concentrations of each drug using 100 *µ*L SH NPs and 50 mmol·L^−1^ KNO_3_. As shown in Table 4, high recovery percentages indicate that the method can be successfully applied with high accuracy for the determination of the interested analytes.

The pharmaceutical dosage form of ATN and AML was evaluated using the developed method. The excellent recovery percent values indicated the potential of the method for determining both analytes in the pharmaceutical dosage forms without suffering from the interferences of the present excipients (Table 5).

To further discover the practical application of the proposed method, a quantitative analysis of the interested drugs in spiked urine samples was done. Two different amounts of known concentration of ATN and AML in mixture were spiked to reach a final concentration of 1 × 10^−4^ mol·L^−1^ for ATN and 5 × 10^−6^ mol·L^−1^ for AML. From the resulted recovery data (Table 5), it can be implied that the method is reliable and could be applied successfully in biological samples.

Table 6 represents a comparison between the proposed method and other previous reported methods that was used for simultaneous determination of ATN and AML. It can be concluded that the proposed method provides higher sensitivity, wider linear range, and lower detection limit with the simplicity of the instrument used comparing with spectroscopic techniques.

## 4. Conclusion

This contribution is considered the first effort to utilize the LSPR resulting from SH NPs prepared by reduction of silver nitrate using hydroxylamine HCL for the simultaneous determination of the studied drugs. The developed method is based on the enhancement effect of plasmon resonance of AML containing four amino groups as attachment points on SH NPs. This effect makes a synergistic behavior resulting in an increase in the sensitivity of ATN and AML determinations. Besides the enhancement effect, this approach carries many advantages of the simplicity and reproducibility as well as low cost application.

From the obtained results, it can be concluded that the proposed method is applied successfully for the analysis of the investigated drugs in their laboratory mixtures, pharmaceutical capsules, and in spiked urine samples.

## Figures and Tables

**Table 1:** Statistical parameters of the proposed method.

Statistical parameter	Atenolol	Amiloride
Linearity range	1 × 10^−5^–1 × 10^−4^ mol·L^−1^	1 × 10^−6^–1 × 10^−5^ mol·L^−1^
Intercept ± SD^a^	0.34 ± 0.0055	0.019 ± 0.0038
Slope ± SD^b^	0.027 ± 9.3 × 10^−4^	0.039 ± 6.4 × 10^−4^
*R* ^2c^	0.9941	0.9986
*R* ^d^	0.9970	0.9993
Mandel test value^e^	7.8 × 10^−9^	7.2 × 10^−8^
LOD (mol·L^−1^)^f^	0.89 × 10^−5^	0.42 × 10^−6^
LOQ (mol·L^−1^)^g^	2.71 × 10^−5^	1.28 × 10^−6^
*S*_*y*_^h^	0.0072	0.0050
*S*_*x*0_^i^	0.0862	0.125
RSD (*n* = 3)^j^	0.178	0.565
Intraday precision^k^	1.94–2.78	1.01–1.67
Interday precision^k^	1.64–3.10	1.25–2.40

^a^Standard deviation of intercept, ^b^standard deviation of slope, ^c^determination coefficient, ^d^correlation coefficient, ^e^tabulated *F* values at *P*=0.95 is 7.7 at (df1 (1), df2 (n-3) = (1,4)), ^f^limit of detection, ^g^limit of quantitation, ^h^standard error, ^i^method standard deviation, ^j^relative standard deviation, and ^k^average of six determinations at three different concentration levels.

**Table 2:** Effect of interferents on the determination of ATL and AML.

Interferention (1 × 10^−3^ mol·L^−1^)	ATN^∗^ concentration found (mol·L^−1^)	AML^•^ concentration found (mol·L^−1^)
	Recovery % ± SDᵒ	Recovery % ± SDᵒ
Ascorbic acid	1.02 × 10^−4^	4.98 ± 10^−6^
	102.0 ± 0.3	99.6 ± 0.2
Na^+^	0.97 × 10^−4^	5.01 × 10^−6^
	97.0 ± 1.2	100.2 ± 0.1
Ni^2+^	1.00 × 10^−4^	4.88 × 10^−6^
	100.0 ± 0.2	97.6 ± 0.5
Ca^2+^	0.99 × 10^−4^	4.99 × 10^−6^
	99.0 ± 0.5	99.8 ± 0.6
NH_4_^+^	0.98 × 10^−4^	5.03 × 10^−6^
	98.0 ± 0.7	100.6 ± 0.1
SO_4_^2−^	1.01 × 10^−4^	5.00 × 10^−6^
	101.0 ± 0.3	100.0 ± 0.3
PO_4_^3−^	0.97 × 10^−4^	4.84 × 10^−6^
	97.0 ± 0.6	96.5 ± 1.0

^∗^ATN concentration is 1 × 10^−4^ mol·L^−1^ (measure at 274 nm). ^•^AML concentration is 5 × 10^−6^ mol·L^−1^ (measure at 364.5 nm). ᵒAverage of three determinations ± SD.

**Table 3:** Robustness results of the proposed method.

Variation	% recoveryᵒ ± SD	
	Atenolol^∗^	Amiloride^•^
No variation^∗∗^	99.0 ± 0.2	97.9 ± 0.8
*Volume of SH NPs*		
98 *µ*L	98.3 ± 0.5	98.3 ± 0.5
102 *µ*L	99.7 ± 0.1	98.2 ± 0.7
*KNO*_*3*_ *conc.*		
49 mmol·L^−1^	100.6 ± 0.8	99.1 ± 0.9
51 mmol·L^−1^	99.3 ± 0.4	100.5 ± 0.3
*Volume taken of the mixed analytes*		
0.9 mL	98.4 ± 0.7	97.8 ± 1.0
1.1 mL	99.1 ± 1.1	100.2 ± 0.4

^∗^ATN concentration is 1 × 10^−4^ mol·L^−1^ (measured at 274 nm). ^•^AML concentration is 5 × 10^−5^ mol·L^−1^ (measured at 364.5 nm). ᵒAverage of three determinations ± SD. ^∗∗^No variation in the experimental conditions of the proposed method.

**Table 4:** Determination of ATN and AML in laboratory-prepared mixtures.

Added amount (mol·L^−1^)		Found amount (mol·L^−1^)		Recovery (%)^#^ ± SD	
ATN^∗^ (×10^−5^)	AML^•^ (×10^−6^)	ATN^∗^ (×10^−5^)	AML^•^ (×10^−6^)		
1	1	0.98	0.99	98.0 ± 1.2	99.1 ± 0.9
2	2	1.92	2.01	96.0 ± 0.8	100.5 ± 0.7
4	4	3.89	3.93	97.3 ± 0.7	98.3 ± 0.4
6	6	6.10	5.88	101.7 ± 0.5	98.0 ± 0.9
8	8	7.79	7.92	97.4 ± 0.4	99.0 ± 0.6
10	10	10.20	9.73	102.0 ± 0.2	97.3 ± 0.1

^∗^ATN is measured at 274 nm. ^•^AML is measured at 364.5 nm. ^#^Average of three determinations ± SD.

**Table 5:** Determination of ATN and AML in the pharmaceutical dosage form and in spiked urine sample.

Product	Drug (content, mg)	Found (mg)			
		Recovery (%)^∗^ ± SD		*F* value	*t* value
		Proposed method	Reported method^a^		
Atenoretic (capsules)	Atenolol, 50	49.8	48.0	3.205	0.233
		99.6 ± 1.2	96 ± 2.0	2.444	0.127
	Amiloride, 2.5	2.40	2.33		
		96.0 ± 0.8	93.2 ± 1.6		

	Spiked amount (mol·L^−1^)	Found amount (mol·L^−1^)			
		Recovery (%)^∗^ ± SD			

Urine sample	Atenolol, 1 × 10^−4^	0.99 × 10^−4^			
		99.0 ± 0.6			
	Amiloride, 0.5 × 10^−5^	0.48 × 10^−5^			
		96.0 ± 1.1			

^∗^Average of three determinations ± SD. ^a^Reference [7].

**Table 6:** A comparison between different approaches used for simultaneous determination of ATN and AML.

Method	Limit of detection (*µ*g·mL^−1^)	Linearity range (*µ*g·mL^−1^)			Ref.
	ATN	AML	ATN	AML	
HPLC	1.38 × 10^−3^	1.4 × 10^−5^	5–50	0.25–2.5	[3]
HPLC	3 × 10^−3^	4 × 10^−3^	40–160	2–8	[4]
Electrophoresis	3 × 10^−3^	4 × 10^−3^	5–250	5–250	[5]
Electrophoresis	0.6	0.5	0.6–10	0.5–10	[6]
UV spectrophotometry	0.111	0.033	15–30	1.25–5	[7]
Spectrofluorimetry	5.9	2.8	5.6–280	10–400	[8]
This method	2.37	0.096	2.66–26.6	0.22–2.29	
